# From innovation to implementation: expanding global access to mechanical circulatory support and heart transplantation

**DOI:** 10.3389/fcvm.2026.1883956

**Published:** 2026-09-11

**Authors:** Laith Alhuneafat, Omar Obeidat, Kevin Buda, Dina Mohammed, Samarth Goyal, Gaspar Del Rio Pertuz, Murad Almasri, Joerg A. Reifart, Adam L. Gottula, Paul Rees, Ilhwan Yeo, Jason Bartos, Tamas Alexy, Demetris Yannopoulos, Andrea M. Elliott

**Affiliations:** 1 Division of Cardiovascular Medicine, University of Minnesota, Minneapolis, MN, United States; 2 Division of Cardiovascular Medicine, University of Texas Medical Branch, Galveston, TX, United States; 3 Department of Critical Care Medicine, Mayo Clinic, Rochester, MN, United States; 4 School of Medicine, University of Jordan, Amman, Jordan; 5 Department of Medicine, University of Minnesota, Minneapolis, MN, United States; 6 Department of Pediatric Cardiology, University of Arkansas, Little Rock, AR, United States; 7 Critical Care Medicine, University of Minnesota, Minneapolis, MN, United States

**Keywords:** cardiogenic shock, mechanical circulatory support, extracorporeal membrane oxygenation, global health, health equity, health systems, heart transplantation, left ventricular assist device

## Abstract

Mechanical circulatory support (MCS) and heart transplantation are established therapies for advanced heart failure and cardiogenic shock. Technological advances have improved outcomes, including randomized evidence supporting the use of temporary MCS, such as percutaneous ventricular assist devices, in selected patients with ST-elevation myocardial infarction–related cardiogenic shock, alongside substantial gains in survival with contemporary durable left ventricular assist devices (LVADs). Despite this progress, access remains highly inequitable. Although global transplant activity has increased in recent years, heart transplantation remains concentrated in a small number of regions; historically, approximately 90% of procedures have been performed in North America and Western Europe. Similarly, temporary MCS use is largely limited to tertiary care centers, while durable LVAD use is concentrated in high-income settings. These disparities reflect differences in health system capacity across infrastructure, workforce, regulation, financing, culture, ethics, longitudinal follow-up, and data systems, as well as variation in health-system investment, language access, health literacy, and the completeness of registry-based data capture. In many resource-limited settings, fragmented care pathways lead to a “bridge to nowhere,” where temporary support is initiated without access to definitive therapy. In this narrative review with scoping methods, we examine global variation in access to MCS and heart transplantation, map patient care pathways, and organize barriers into key system domains. We further synthesize potential solutions and propose a practical implementation roadmap spanning regionalized shock systems, durable MCS referral and follow-up networks, donor management infrastructure, workforce development, health financing strategies, and registry-based quality improvement.

## 1 Introduction

Mechanical circulatory support (MCS) and heart transplantation are cornerstone therapies for cardiogenic shock and advanced heart failure, yet access to these life-saving interventions remains highly inequitable across regions (1–4). These therapies span acute and chronic clinical scenarios, including acute myocardial infarction–related cardiogenic shock, fulminant myocarditis, post-cardiotomy shock, and chronic end-stage heart failure refractory to medical therapy (5–7). Heart transplantation remains the gold standard for selected patients with irreversible advanced heart failure, but its availability is limited by donor scarcity, specialized infrastructure, and the need for durable post-transplant follow-up (5–7). Reliance on MCS has increased substantially, driven by a persistent mismatch between organ supply and demand, prolonged wait times, improved device outcomes, expanded regulatory approval for destination therapy, and evolving transplant allocation policies (8–10).

MCS encompasses both temporary and durable platforms. Temporary MCS includes intra-aortic balloon pumps (IABPs), extracorporeal membrane oxygenation (ECMO), and percutaneous ventricular assist devices (pVADs), which provide short-term hemodynamic support across acute cardiovascular settings (11–13). Durable MCS devices, principally left ventricular assist devices (LVADs), biventricular assist devices (BiVADs), and total artificial hearts (TAHs), are surgically implanted for longer-term support and may serve as a bridge to transplantation, bridge to recovery, or destination therapy for selected patients ineligible for transplantation (14–16).

Technological advances have substantially improved outcomes. Contemporary durable LVADs now achieve approximately 58% five-year survival, while randomized trials have demonstrated benefit of temporary MCS in specific high-risk populations, including selected patients with ST-elevation myocardial infarction-related cardiogenic shock and refractory out-of-hospital cardiac arrest (1, 4, 17, 18). Despite these advances, global access to MCS and heart transplantation remains profoundly inequitable, with most procedures concentrated in the United States and Western Europe and much of the world lacking access to therapies now routine in many high-income settings (19–22). Even temporary MCS, which is more broadly distributed, remains limited by tertiary infrastructure, trained personnel, and regional transfer systems (23).

Critically, the barriers to access are not primarily technological. Rather, they are systemic: inadequate financing and reimbursement structures, workforce shortages, regulatory variability, limited infrastructure, cultural barriers to organ donation, language and health-literacy barriers, limited longitudinal follow-up capacity, and the absence of registries and quality systems (5, 19, 24). These system-level constraints determine whether device innovation translates into patient benefit. Even within high-income countries, persistent disparities by race, sex, socioeconomic status, geography, language access, health literacy, and cultural context limit equitable access to advanced heart failure therapies (2, 3, 25). In this review, we examine global variation in access to MCS and heart transplantation through an implementation-focused health systems lens, emphasizing infrastructure, workforce, financing, regulation, culture and ethics, equity, referral pathways, and data systems. We map patient care pathways, organize barriers into key domains, and synthesize potential solutions to inform a roadmap for expanding equitable access worldwide, while recognizing that global comparisons rely on heterogeneous registries, reporting years, and health-system contexts.

## 2 Methods

We conducted a narrative review using a structured search strategy. MEDLINE (PubMed), Embase, Scopus, and Web of Science were searched for studies published between January 2015 and February 2026. An updated targeted search was performed on May 4, 2026, to identify recently published registry reports, guidelines, policy documents, and other high-relevance sources published or updated after the initial search window. We included clinical guidelines, registry reports, observational studies, systematic reviews, and meta-analyses addressing access to mechanical circulatory support and heart transplantation. Editorials, commentaries, letters, conference abstracts, animal studies, single-patient case reports, and bench-test studies were excluded. No language restrictions were applied during the search; however, the review was limited to studies available in English. Given the global and systems focus of this review, additional sources such as professional society reports, registry data, and policy documents were included when relevant. Findings were synthesized narratively and organized into key system domains. Key barrier domains were identified iteratively through thematic synthesis of the literature. Because this review synthesizes evidence from registries, annual reports, observational cohorts, policy documents, and studies conducted across different years and health systems, cross-country comparisons should be interpreted cautiously. Data sources differed in reporting periods, denominator definitions, device categories, submission requirements, and follow-up completeness; therefore, metrics such as procedures per million population, LVAD implant volume, ECMO center density, and transplant rates were used to describe broad access patterns rather than directly comparable health-system performance.

## 3 Global landscape of MCS and heart transplantation

### 3.1 Heart transplantation

In 2024, the Global Observatory on Donation and Transplantation (GODT) recorded 10,287 heart transplants in 59 countries, representing 5.9% of 173,727 solid organ transplants worldwide (20). Access to heart transplantation remains concentrated in a small number of countries and health systems. At the WHO regional level, the Americas and Europe accounted for approximately 40% and 26% of all solid organ transplants, respectively, whereas the Eastern Mediterranean contributed only 2% and Africa less than 1% (20). A comprehensive review of global heart transplant activity reported that more than 7,000 heart transplants were performed annually by 2021, with approximately 90% occurring in the United States and Western Europe between 2010 and 2018. (19) These estimates should be interpreted as broad indicators of geographic concentration because reporting periods and registry structures differ across data sources.

Country-level data illustrate the magnitude of disparity. Heart transplant rates reach 12–14 per million population (pmp) in the United States, 9.3 pmp in Croatia, and 6.6 pmp in Spain, but fall to 3.3 pmp in South Korea, 1.7 pmp in Brazil, 0.6 pmp in Japan, 0.5 pmp in China, and 0.17 pmp in India (19, 20). Notably, these disparities are not explained by national income alone. Japan, despite having one of the world's most advanced healthcare systems, reports only 0.6 transplants per million whereas Croatia exceeds 9 per million (19, 26). In Latin America, transplant activity is present but fragmented: Brazil performs approximately 350 heart transplants per year (1.7 pmp), compared with only 33 per year in Mexico (0.3 pmp) (20, 27). In Sub-Saharan Africa, outside of South Africa (0.7 pmp), there are effectively no active heart transplant programs, translating to approximately 0.09 pmp across the region (19, 20, 28, 29). In India, which surpassed China in 2023 to become the world's most populous country, only 102 heart transplants were performed that year (30).

A recent longitudinal analysis of GODT data from 2008 to 2023 confirmed that while global transplant activity has increased substantially, gains have been concentrated in countries with very high Human Development Index scores (31). In other words, the absolute number of transplants has increased globally, but growth has occurred disproportionately in countries that already had greater health-system capacity. The Slope Index of Inequality, a population-level measure of the absolute difference in transplant rates between higher- and lower-development countries, widened from 55.9 to 73.9 (31). The estimated global heart transplant capacity gap exceeds 30,000 procedures annually (31).

### 3.2 Durable mechanical circulatory support

Durable LVADs are now established as either a bridge to transplantation or destination therapy in many high-income countries, with contemporary outcomes approaching those of heart transplantation in selected populations (4, 17). In the United States, approximately 3,000 primary LVAD implants are performed annually across specialized centers, with virtually all newly implanted devices being fully magnetically levitated (HeartMate 3) (21, 32). Following the 2018 UNOS allocation revision, use of LVADs as a bridge to transplant declined to below 15% in the United States, while destination therapy now represents the majority of implants (21, 33).

Within Europe, reported LVAD implantation rates show substantial variation. Countries such as Greece, Portugal, Spain, and Italy report rates at or below 2 pmp, whereas Austria, Germany, the Netherlands, and Switzerland range between 4 and 9 pmp (22). These differences persist despite broadly similar epidemiological profiles of advanced heart failure, suggesting that access is shaped by the availability and distribution of implanting centers, national reimbursement policies, and the structure of longitudinal post-implant care rather than by clinical need alone (22).

Outside high-income countries, available data suggest that durable MCS access is extremely limited. Most implants occur in North America and Western Europe, with far fewer in Asia–Pacific and Latin America and only isolated reports from Africa (34–36). In middle-income countries such as Brazil, Chile, and Serbia, LVAD programs are confined to a few centers and often rely on philanthropy, private insurance, or intermittent government support (34, 37). Infrastructure density reflects this disparity; for example, ESC Atlas data show approximately 0.4 LVAD-implanting hospitals per million population in high-income countries vs. 0.1 in middle-income settings (36, 38). In India, only 128 cumulative LVAD implants had been performed through March 2023 in a population exceeding 1.4 billion, compared with approximately 2,500–3,000 implants performed annually in the United States, which has a population of approximately 330 million (30, 32).

### 3.3 Temporary mechanical circulatory support

Temporary MCS, including IABPs, pVADs, and ECMO, is more widely available than durable LVAD therapy but remains closely tied to tertiary care capacity and regional referral networks (23). The Extracorporeal Life Support Organization (ELSO) registry has documented 154,568 ECMO runs across 780 centers in 57 countries between 2009 and 2022, with annual adult ECMO volume increasing from 851 cases in 2009 to nearly 18,000 in 2021; however, the 2020–2021 surge was largely attributable to COVID-19–related respiratory ECMO rather than cardiac indications (39). By 2023, over 750 ELSO-registered centers were operational globally (39). Because some active ECMO programs do not participate in ELSO reporting, these registry data likely underestimate total ECMO capacity. The growth of ELSO-registered centers nevertheless illustrates how rapidly ECMO can expand when infrastructure, trained teams, and reimbursement pathways are established.

The IABP, historically central to cardiogenic shock management, has declined in relative use following evidence showing limited mortality benefit, yet remains the most widely used form of temporary support worldwide, with more than 200,000 procedures annually (3, 40–43). Its persistence reflects low cost, bedside deployability, and a favorable safety profile, making it the most accessible MCS modality in resource-limited settings (40). In contrast, percutaneous VAD use, particularly the Impella platform (Impella CP, Johnson & Johnson, New Brunswick, NJ), has expanded substantially in higher-income systems. The DanGer Shock trial demonstrated a 12.7% absolute mortality reduction at 180 days among appropriately selected patients with ST-elevation myocardial infarction-related cardiogenic shock, contributing to renewed guideline and expert consensus support for selective use of microaxial flow pump support in this populatio (1, 44, 45). However, higher complication rates (bleeding, vascular injury) and device cost limit widespread adoption (1).

Geographic distribution of temporary MCS remains uneven. Most ECMO and advanced pVAD activity is concentrated in North America and Western Europe, with growing but limited expansion in Asia–Pacific, Latin America, and South-West Asia (39–41). In the United States, a recent geospatial analysis found that approximately 67% of the population lives within 45 min of an ECMO-capable center, but access is significantly lower among Hispanic, elderly, rural, and low-income populations (46). In many low- and middle-income countries, there are no ELSO-registered centers, and where ECMO exists it is typically confined to one or a few tertiary hospitals (39). In Latin America, IABP is often the only widely available MCS option, while more advanced platforms remain restricted by cost and training requirements (37). In contrast, some higher-resource Asian systems have historically relied more heavily on ECMO within transplant pathways; for example, Korean Organ Transplant Registry data show that ECMO was used as a bridge to transplant in 28.9% of heart transplant recipients in South Korea, compared with approximately 7% in the contemporary U.S. UNOS era (47, 48). This pattern likely reflects differences in waitlist urgency, donor availability, allocation policy, insurance coverage, device approval, and availability of alternative MCS platforms, rather than resource availability alone.

### 3.4 Program classification

**Figure 1:**
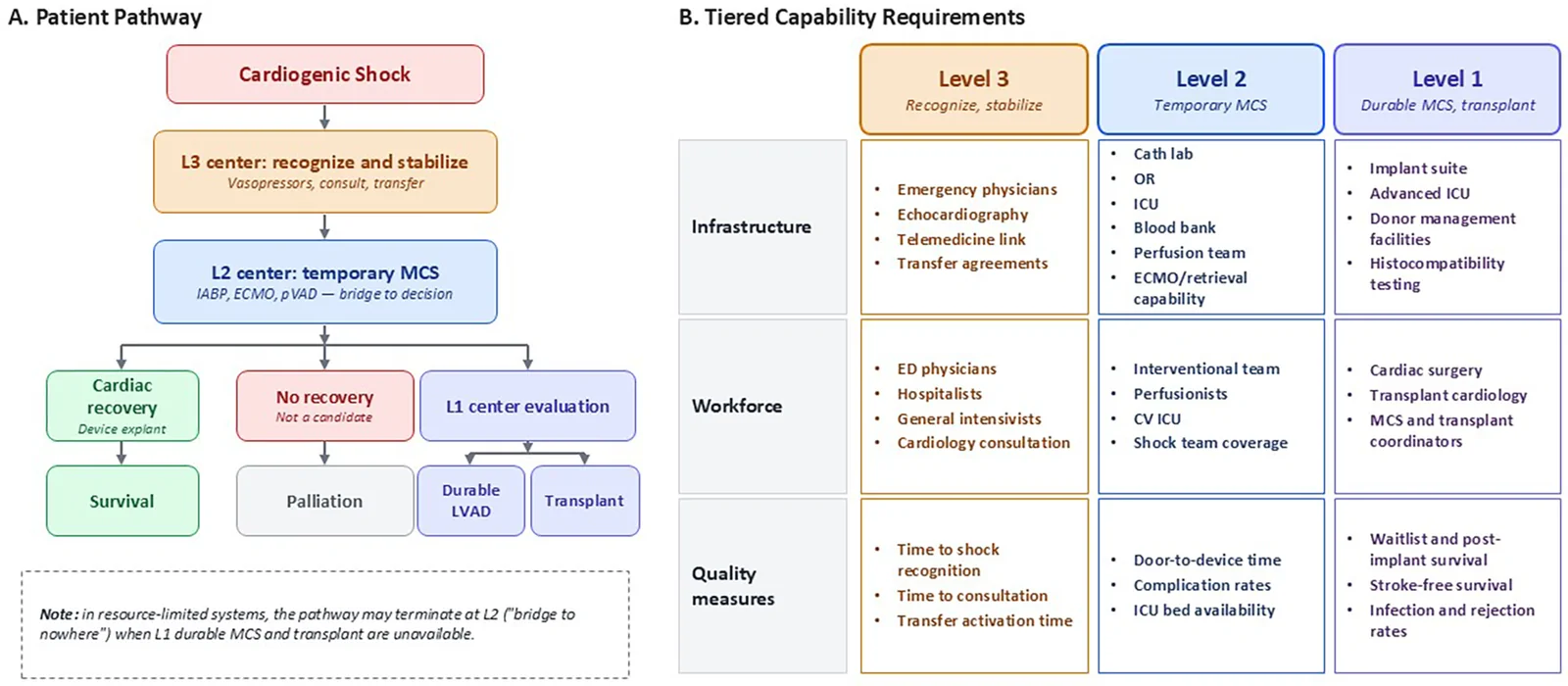
Patient pathway and tiered center capability requirements for mechanical circulatory support and heart transplantation. Panel A illustrates a simplified pathway from cardiogenic shock recognition to temporary mechanical circulatory support, recovery, durable left ventricular assist device therapy, heart transplantation, or palliation. Panel B summarizes infrastructure, workforce, and quality measures across Level 3 frontline centers, Level 2 temporary mechanical circulatory support centers, and Level 1 durable mechanical circulatory support and transplant centers. CV, cardiovascular; ECMO, extracorporeal membrane oxygenation; ED, emergency department; ICU, intensive care unit; IABP, intra-aortic balloon pump; LVAD, left ventricular assist device; MCS, mechanical circulatory support; OR, operating room; pVAD, percutaneous ventricular assist device.

To provide a descriptive framework for the capabilities required at each level of the care pathway, we adopt the three-tier center classification endorsed in the 2025 American College of Cardiology Expert Consensus on cardiogenic shock management (44). Level 3 centers are frontline facilities capable of identifying and stabilizing cardiogenic shock and activating rapid consultation and transfer. Level 2 centers can implant and maintain temporary MCS, with monitored beds, blood bank access, cardiac catheterization laboratory and operating room capacity, and trained perfusion teams. Level 1 centers offer durable MCS and heart transplantation, with donor management interfaces, advanced ICU and operative capacity, and retrieval teams (11, 44, 49). This tiered structure provides a practical framework for matching regional capabilities to patient needs across the continuum from shock recognition to definitive therapy (Figure 1).

This classification provides a common language for describing regional capabilities, identifying gaps, and aligning quality measurement across the care pathway, while allowing metrics to be adapted to local data infrastructure and reporting capacity. At Level 3 centers, relevant process measures may include time to shock recognition, time to escalation consultation, and time to transfer activation. At Level 2 centers, measures may include time from decision to temporary MCS initiation, device availability, complication rates, and ICU capacity. At Level 1 centers, quality measures extend to waitlist outcomes, post-implant and post-transplant survival, stroke-free survival, infection rates, and successful transition from temporary support to recovery, durable MCS, transplantation, or palliation.

### 3.5 Patient pathways: from shock to definitive therapy

The clinical trajectory for patients requiring MCS, whether for AMI-related cardiogenic shock, acute myocarditis, post-cardiotomy failure, or decompensated chronic end-stage heart failure presenting with shock, is rarely linear. Instead, it involves a series of decision points at which patient physiology and system capabilities must align. Most patients follow a pathway from cardiogenic shock recognition through stabilization, temporary MCS support, and ultimately to one of three outcomes: native heart recovery, transition to durable therapy where available (LVAD or transplant), or palliative care (11, 49).

The pathway begins with prompt recognition of cardiogenic shock, most commonly at frontline (Level 3) centers that lack MCS capability. Patients requiring transfer to specialized hubs experience higher mortality when transfer is delayed, making early identification of those most likely to benefit from escalation essential (49, 50). At Level 2 centers, which maintain temporary MCS capability with established perfusion teams, 24/7 catheterization laboratory and operating room access, and dedicated intensive care units, the treatment goal shifts to stabilization and bridge-to-decision. At this stage, delays in temporary MCS initiation may worsen outcomes in selected patients; observational data and meta-analyses suggest that earlier initiation is associated with improved survival in acute myocardial infarction-related cardiogenic shock (50, 51).

Following temporary MCS initiation, three pathways are possible. First, some patients achieve native heart recovery and are suitable for device explant. Second, patients who do not recover and are ineligible for durable therapies require structured goals-of-care discussions regarding palliation or withdrawal of life-sustaining therapies. Third, patients who do not recover but remain locally eligible candidates for durable therapies require evaluation for LVAD implantation or heart transplantation at Level 1 centers (Figure 1) (11, 49).

Pathway performance varies dramatically by region. In well-organized systems with established hub-and-spoke networks, early shock recognition at Level 3 centers, coordinated transfer, rapid device initiation, and seamless escalation to durable therapies produce favorable outcomes. Centers that employ dedicated multidisciplinary shock teams have demonstrated shorter door-to-device times, more appropriate device selection, and improved in-hospital and short-term survival (44, 52–54). Contemporary transplant programs report one-year survival approaching 90%, and contemporary durable LVAD therapy now achieves approximately 58% five-year survival (16, 17, 55).

In contrast, fragmented or resource-limited systems often disconnect temporary MCS from access to durable therapies, creating a potential “bridge to nowhere.” In parts of Asia, Africa, and Latin America, ECMO or IABP may be available in tertiary centers, but durable LVAD programs and transplant services are limited or absent (34–37). Patients may initially stabilize on temporary support but have no pathway to definitive therapy if they fail to achieve sufficient cardiac recovery with or without inotropic support. This phenomenon often reflects systemic failure rather than individual clinical decisions: late initiation of support, absent referral pathways, limited ICU capacity, high device costs, low donor availability, and the absence of structured allocation systems all contribute (5, 19, 49). Prolonged temporary MCS without a defined exit strategy is thought to increase the risk of complications, including bleeding, thrombosis, infection, and limb ischemia, with potential downstream effects on outcomes and resource utilization (56, 57).

## 4 Barriers to access

**Figure 2:**
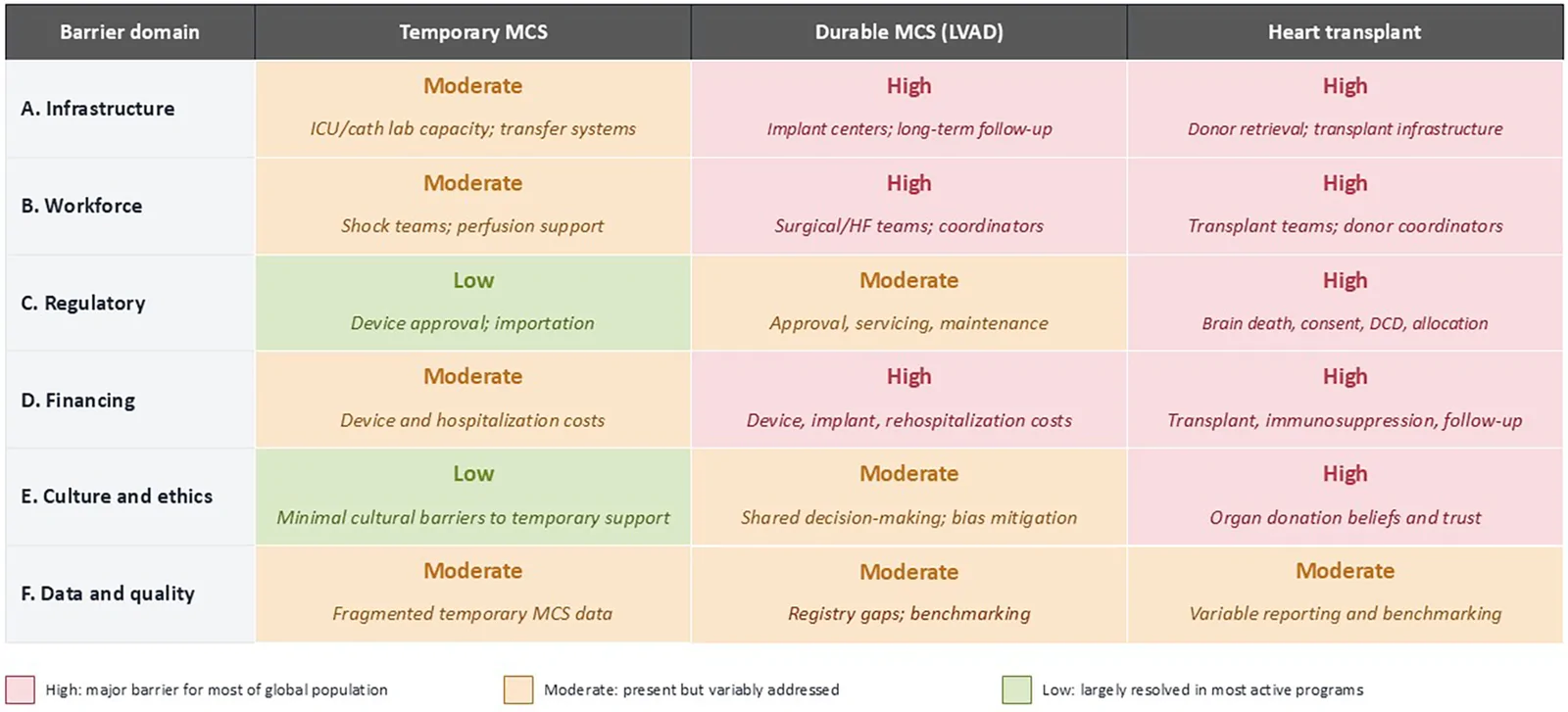
Heat map of access barriers across mechanical circulatory support and heart transplantation. Relative severity of access barriers across temporary mechanical circulatory support, durable mechanical circulatory support, and heart transplantation. Higher ratings indicate a larger gap between current global capacity and equitable access for most of the global population. ECMO, extracorporeal membrane oxygenation; IABP, intra-aortic balloon pump; LVAD, left ventricular assist device; MCS, mechanical circulatory support.

The regional disparities described above are not explained by differences in disease burden or device availability alone, but rather reflect underlying health system constraints that shape access across the continuum of care. The barriers to global access to MCS and heart transplantation are multifactorial and interconnected, spanning infrastructure, workforce, regulation, financing, culture and ethics, equity, and data systems. We organize these barriers into six domains, each of which operates differently across temporary MCS, durable LVAD therapy, and heart transplantation. The relative severity of these barriers is summarized in a heat map to illustrate how access constraints differ by therapy type (Figure 2).

### 4.1 Infrastructure and logistics

MCS and heart transplantation require a layered, coordinated infrastructure that extends well beyond the device itself. Temporary MCS demands intensive care unit beds with hemodynamic monitoring, on-site cardiac catheterization laboratories and operating rooms, reliable blood banking, and perfusion support. Durable LVAD programs add the need for dedicated implant suites, specialized post-operative recovery units, and long-term ambulatory follow-up infrastructure. Heart transplantation further requires organ procurement organizations, donor management facilities, retrieval and transportation logistics, and histocompatibility and immunologic testing capacity to support donor-recipient matching and post-transplant monitoring (11, 23, 58).

The distribution of cardiac surgical infrastructure, which is essential for both MCS and transplantation, is highly uneven across regions. Global capacity remains limited, with a median of 0 (0–0.06) centers per million population in low-income countries compared with 0.75 (0–1.44) in high-income settings, and even when adjusted to disease burden, the number of centers falls far short of estimated need across all income groups (58). In the United States, approximately two-thirds of the population lives within a 45 min drive of an ECMO-capable center, but geographic access is significantly reduced for rural and some underserved populations (46). In Sub-Saharan Africa, 31 of 54 countries have cardiac surgery programs, yet surgical volume remains profoundly limited, at approximately 0.5 procedures per million population in Nigeria compared with 142 per million in South Africa and over 1,200 per million in Germany (29, 59). In Southeast Asia, the cardiac surgical workforce remains severely constrained, with extremely low surgeon-to-population ratios and several countries (e.g., Bhutan, Brunei, Laos, and Timor-Leste) lacking any cardiac surgical capacity (60, 61).

Transfer and retrieval networks are critical for ensuring that patients at Level 3 centers can access temporary MCS, durable MCS, transplantation, or palliation in a timely fashion (62). In many low- and middle-income countries, the absence of coordinated transfer systems, including mobile retrieval capability, limits geographic access to advanced therapies. As a result, interfacility transfer for cardiogenic shock is often associated with significant delays and excess mortality (49, 50).

### 4.2 Workforce and training

Sustainable MCS and transplant programs require multidisciplinary teams spanning cardiac surgery, heart failure and interventional cardiology, critical care, perfusion, program coordination, and specialized nursing disciplines. They also require outpatient LVAD and transplant nursing teams, pharmacists, rehabilitation specialists, social workers, and coordinators who can support longitudinal care after discharge. The global distribution of this workforce is profoundly unequal. High-income countries average 7.15 cardiac surgeons per million population compared with 0.04 per million in low-income countries, representing a nearly 180-fold disparity (61).

Perfusionist shortages are particularly acute. Even in the United States, where approximately 4,000 certified perfusionists support cardiac surgical and extracorporeal programs, workforce surveys demonstrate persistent vacancy rates exceeding 10% and substantial turnover (63). Workforce sustainability is further constrained by high rates of burnout across cardiovascular care teams, particularly in high-intensity environments such as cardiac intensive care and shock programs. Survey data demonstrate burnout affecting up to 40%–59% of cardiology medical staff, with associated intent to reduce clinical effort or leave practice (64). For MCS programs specifically, 24/7 on-call requirements demand robust call rosters balancing burnout prevention (typically no more than 1:4 or 1:5 call frequency) with adequate case volume to maintain operator competency (at least 15–20 cases annually per operator) (11). Programs that depend on a single “MCS champion” or a small number of highly specialized clinicians are particularly vulnerable, because unplanned absences, turnover, or burnout may suspend services entirely.

Workforce limitations extend beyond workforce density to include gaps in training, awareness, and retention. Migration of trained specialists from low- and middle-income countries to higher-resource settings may further exacerbate workforce shortages and limit program development (65). Low procedural volume can also create a self-reinforcing cycle: limited case numbers make it difficult to train and retain specialized teams, while the lack of specialized teams further restricts program growth and referral confidence. Beyond workforce availability and program operations, effective access also depends on timely recognition and referral. In many regions, limited awareness of advanced heart failure therapies among both patients and referring clinicians contributes to delayed recognition and referral, restricting access to MCS and transplantation (66, 67). These barriers may be amplified when educational materials, referral pathways, and specialist consultations are not accessible to patients or clinicians in their preferred language. Delays in referral and specialist evaluation are associated with worse outcomes in heart failure, including increased mortality with longer time to cardiology consultation (66).

### 4.3 Regulatory and legal frameworks

Regulatory pathways for medical device approval vary substantially across jurisdictions and directly influence access to MCS technologies. In the United States, high-risk devices such as LVADs require Premarket Approval (PMA) through the FDA, with mean decision times of approximately 230 days (68). Only 12.3% of the 1,041 products that received Breakthrough Device designation between 2015 and 2024 progressed to marketing authorization (68). The European Union's transition to the Medical Device Regulation (MDR) in May 2021 introduced stricter clinical evidence requirements and increased regulatory timelines, creating particular challenges for smaller manufacturers and novel device iterations (68). In many low- and middle-income settings, regulatory capacity is limited or fragmented, with reliance on external approvals and variable national processes that may delay access to new technologies (69). Beyond initial approval, importation rules, servicing requirements, and the availability of maintenance contracts may further limit safe program expansion, particularly in countries reliant on imported devices (34, 37). South Korea illustrates this interaction between regulation and reimbursement: limited durable LVAD coverage before 2018 contributed to greater historical reliance on ECMO as a bridge to transplantation, while access to newer pVAD platforms has depended on subsequent government approval pathways (47, 70).

The 2020 World Brain Death Project established minimum clinical standards for brain death determination and highlighted substantial global variability in implementation, with prior survey data demonstrating marked disparities between high- and low-income countries (97% vs. 22% with institutional protocols) (71, 72). Donation after circulatory death (DCD) has emerged as an important strategy to expand the donor pool. In the United States, DCD hearts now account for approximately 14% of heart transplants, up from 3.4% in 2020 (55); however, legislation and clinical practice surrounding DCD vary considerably across jurisdictions. Adoption of DCD heart transplantation is constrained by the need for specialized ex-vivo perfusion technologies (e.g., normothermic perfusion systems) (73), which require regulatory approval and reimbursement pathways that are absent in many countries.

Consent models for organ donation also differ: opt-out (presumed consent) systems are used in Spain, France, and the Netherlands, while opt-in systems predominate in the United States, Germany, and Japan (74). However, recent evidence challenges the assumption that opt-out policies independently increase donation rates; a longitudinal analysis of five countries that switched to opt-out found no significant increase in organ donation rates without complementary investments in procurement infrastructure and public awareness (74, 75). These findings suggest that consent policy should be viewed as one component of a broader donation system rather than as an isolated intervention, with public trust, family communication, and procurement capacity shaping whether legal consent frameworks translate into actual donor authorization and organ recovery. Donor allocation frameworks also vary across countries and may influence equity, waitlist outcomes, and organ transport logistics (19, 76).

### 4.4 Financing

The cost of MCS devices and the infrastructure required to support them represent a fundamental barrier to access, particularly in resource-limited settings. Contemporary analyses indicate that LVAD implantation and early care are associated with high costs across health systems, with European data demonstrating costs exceeding approximately $130,000 USD (≈£100,000) and substantial additional expenditures from hospitalization and longitudinal care (77, 78). In Singapore, a recent cost-effectiveness analysis of HeartMate 3 LVAD therapy for transplant-ineligible patients with end-stage heart failure estimated total per-person costs of approximately US $371,000, with incremental costs of approximately US $302,000 compared with optimal medical management (78). Cost-effectiveness analyses across diverse health systems have generally found that LVADs as destination therapy exceed conventional willingness-to-pay thresholds, although newer-generation devices have improved the cost-effectiveness profile in selected populations (77, 78). Financing barriers extend beyond the purchase price of the device: program viability depends on whether reimbursement covers disposables, maintenance, complications, rehospitalizations, outpatient visits, medications, rehabilitation, caregiver support, and long-term follow-up, rather than implantation alone (77, 78).

At the health-system level, affordability is also shaped by the proportion of national resources allocated to health care, commonly measured as current health expenditure as a percentage of gross domestic product, and by how those resources are distributed across public coverage, private insurance, out-of-pocket spending, capital investment, and longitudinal specialty care (79). Countries within the same income category may therefore differ substantially in their capacity to sustain LVAD programs, temporary MCS readiness, transplant evaluation, donor procurement, and post-discharge follow-up. Incorporating health-expenditure allocation alongside device-level costs may better explain why advanced heart failure infrastructure and long-term cardiovascular outcomes vary across health systems.

In the United States, CMS reimbursement for LVAD therapy has historically been contingent on participation in the INTERMACS registry, linking payment to registry-based reporting and establishing a de facto quality standard for LVAD programs (77, 80). This illustrates how reimbursement can be linked to data submission and quality oversight, although similar mechanisms are often absent for temporary MCS and in many lower-resource settings. In the United Kingdom, LVADs as destination therapy are not routinely commissioned within the National Health Service, reflecting uncertainty regarding cost-effectiveness and resulting in restricted access to therapy (77). In Brazil, durable MCS is not covered by the public health system (SUS), and existing LVAD programs rely on philanthropy and private insurance (34). In India, LVAD implantation costs are substantially lower than in Western countries but remain prohibitive for most patients given the overall constraints in health-system financing and financial protection.

Comparable cost data for temporary MCS demonstrate similarly high resource utilization. In the United States, ECMO and percutaneous support devices are associated with hospitalization costs often exceeding $140,000–$190,000 per episode, underscoring the substantial system-level financial burden of temporary support therapies (79). Device pricing and supply costs may also vary substantially across markets, reflecting differences in procurement volume, negotiated pricing, importation costs, maintenance contracts, and manufacturer market strategies. Lower-volume programs may face additional procurement disadvantages, including limited negotiating power, inconsistent access to maintenance contracts, and stockout risk for consoles, pumps, cannulas, disposables, and replacement components (34, 58).

### 4.5 Culture, ethics, and equity

Cultural and religious attitudes toward organ donation and advanced medical technology represent important but modifiable barriers to access. While official positions of many major world religions are broadly compatible with organ donation, theological interpretation and practical willingness vary substantially within and across traditions (81). In Islam, although organ donation is widely considered permissible by many scholars and the 1986 Conference of Islamic Jurists accepted brain death as a criterion for death determination, uncertainty regarding religious permissibility and variability in interpretation remain important barriers in many Muslim-majority settings (82). Among Indian populations, many of whom identify with Hindu traditions, beliefs regarding death, reincarnation, and funeral practices may influence attitudes toward organ donation, with concerns about body integrity and ritual practices contributing to variability in willingness (83). In Japan, a deeply rooted cultural concept of death tied to the cessation of heartbeat, combined with historical distrust following the 1968 Wada heart transplant controversy, has resulted in one of the lowest organ donation rates among high-income countries (19, 26). Across contexts, family beliefs, health literacy, trust in medical institutions, and communication around brain death and donation can strongly influence whether donation is accepted in practice, even when formal religious or legal frameworks permit it.

Equity disparities in access to MCS and transplantation persist even within well-resourced health systems. In the United States, population-level rates of LVAD implantation and heart transplantation per 1,000 adults with HFrEF are substantially lower among women and Black men compared with White men, with reductions of up to 72% for LVAD use in women and 25%–46% for LVAD and transplantation among Black men (84). Socioeconomic factors further shape access: patients from socioeconomically distressed communities have higher waitlist mortality and worse post-transplant survival, while rural residence is associated with lower rates of waitlist registration and transplantation (2).

In the United States, language access, health literacy, and cultural concordance are additional determinants of equity in durable MCS and heart transplantation. Limited English proficiency and low health literacy may delay advanced heart failure referral, impede transplant or durable MCS education, complicate psychosocial and caregiver assessment, and affect adherence to device care, anticoagulation, immunosuppression, and longitudinal follow-up (85, 86). Programs should therefore incorporate professional interpreter services, plain-language and translated education materials, teach-back, culturally responsive counseling, and documentation of language needs across referral, candidacy assessment, and long-term care.

Multidisciplinary candidate selection for MCS and transplantation is necessary to balance clinical, psychosocial, and ethical considerations, but may introduce subjectivity and disadvantage patients with limited social support, unstable insurance coverage, transportation barriers, or difficulty meeting intensive follow-up requirements. Moreover, limited patient awareness of advanced heart failure and available therapies further contributes to delayed presentation, with individuals often seeking care at stages that limit eligibility for MCS or transplantation (36).

### 4.6 Data and quality systems

Globally, registry participation remains fragmented. The IMACS registry, the only international durable MCS registry, was inactive from 2019 to 2024 due to data-sharing constraints related to the EU General Data Protection Regulation, resulting in a gap in global outcome surveillance (87). Although its relaunch in 2025 represents progress, international benchmarking remains limited (87).

The ISHLT International Thoracic Organ Transplant Registry represents a complementary international benchmarking platform, incorporating data from multiple global partners and providing longitudinal insights into transplant practice; however, it has also faced interruptions in data collection related to evolving privacy and data-sharing regulations (76). In many low- and middle-income countries, national registries for MCS and transplantation are absent, and participation in international registries remains incomplete and uneven, as these systems rely on voluntary data submission and are constrained by cost, limited data infrastructure, workforce capacity, and regulatory barriers to data sharing (5, 30). As a result, reported MCS and transplant volumes should be interpreted cautiously, particularly in low-resource settings where incomplete data may underestimate unmet need.

## 5 Solutions and improvement roadmap

**Figure 3:**
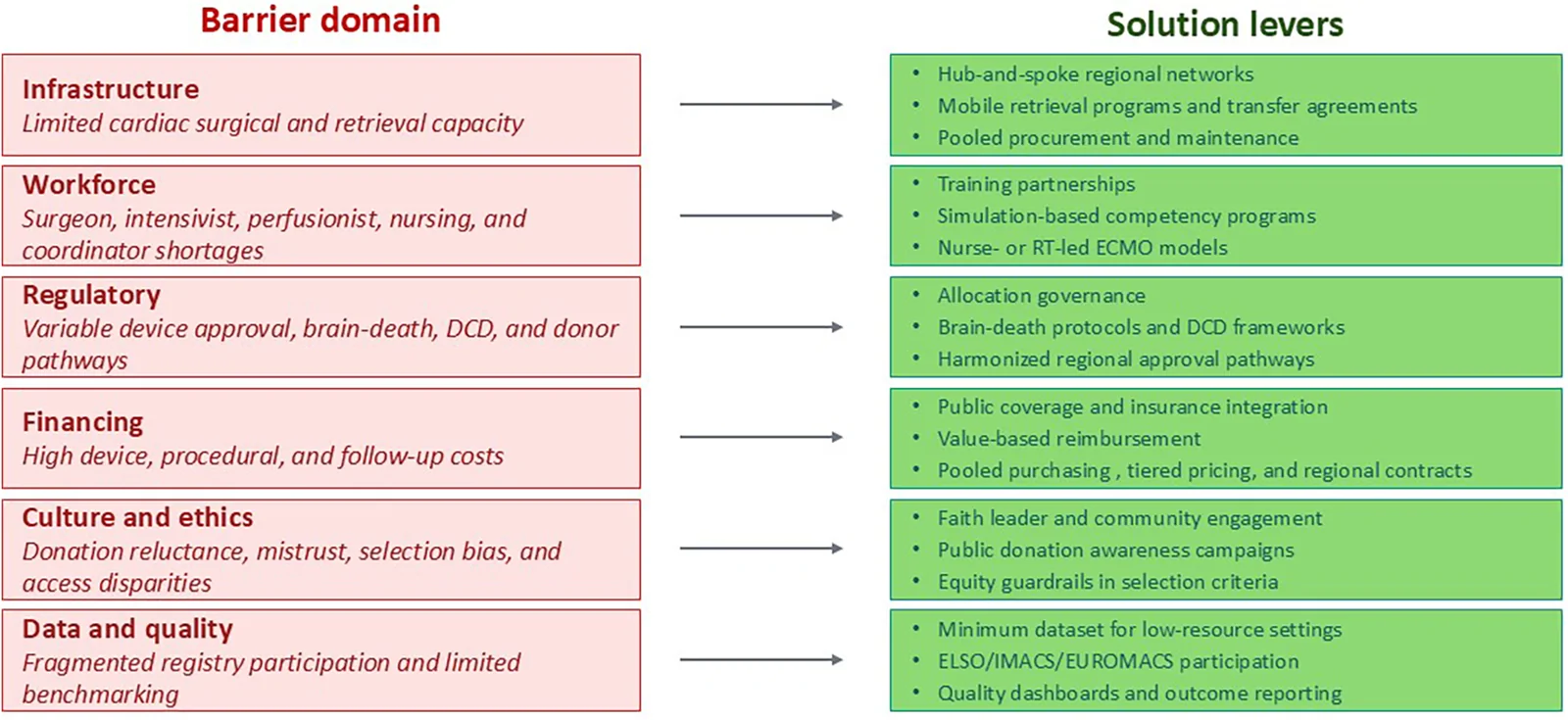
Barriers to global access and corresponding solution levers for mechanical circulatory support and heart transplantation. Major barrier domains are paired with practical implementation strategies that may expand equitable access across temporary mechanical circulatory support, durable mechanical circulatory support, and heart transplantation. DCD, donation after circulatory death; ECMO, extracorporeal membrane oxygenation; ELSO, Extracorporeal Life Support Organization; EUROMACS, European Registry for Patients with Mechanical Circulatory Support; IMACS, International Society for Heart and Lung Transplantation Mechanically Assisted Circulatory Support Registry; MCS, mechanical circulatory support; RT, respiratory therapist.

Addressing the systemic barriers to global access requires coordinated action across five interdependent lever domains: technology, system design and governance, operational sustainability, training, and data infrastructure. These solution levers correspond directly to the major barriers described above, including infrastructure, workforce, regulatory, financing, cultural, ethical, equity, and data constraints (Figure 3). We outline interventions within each domain and propose a practical roadmap for expanding access while maintaining quality, safety, and equity. Across these domains, the highest-priority actionable recommendations are to regionalize shock recognition and transfer pathways, develop durable MCS referral and follow-up networks, strengthen donor management and retrieval infrastructure, align financing with long-term program sustainability, and build minimum datasets for benchmarking and equity monitoring.

**Figure 4:**
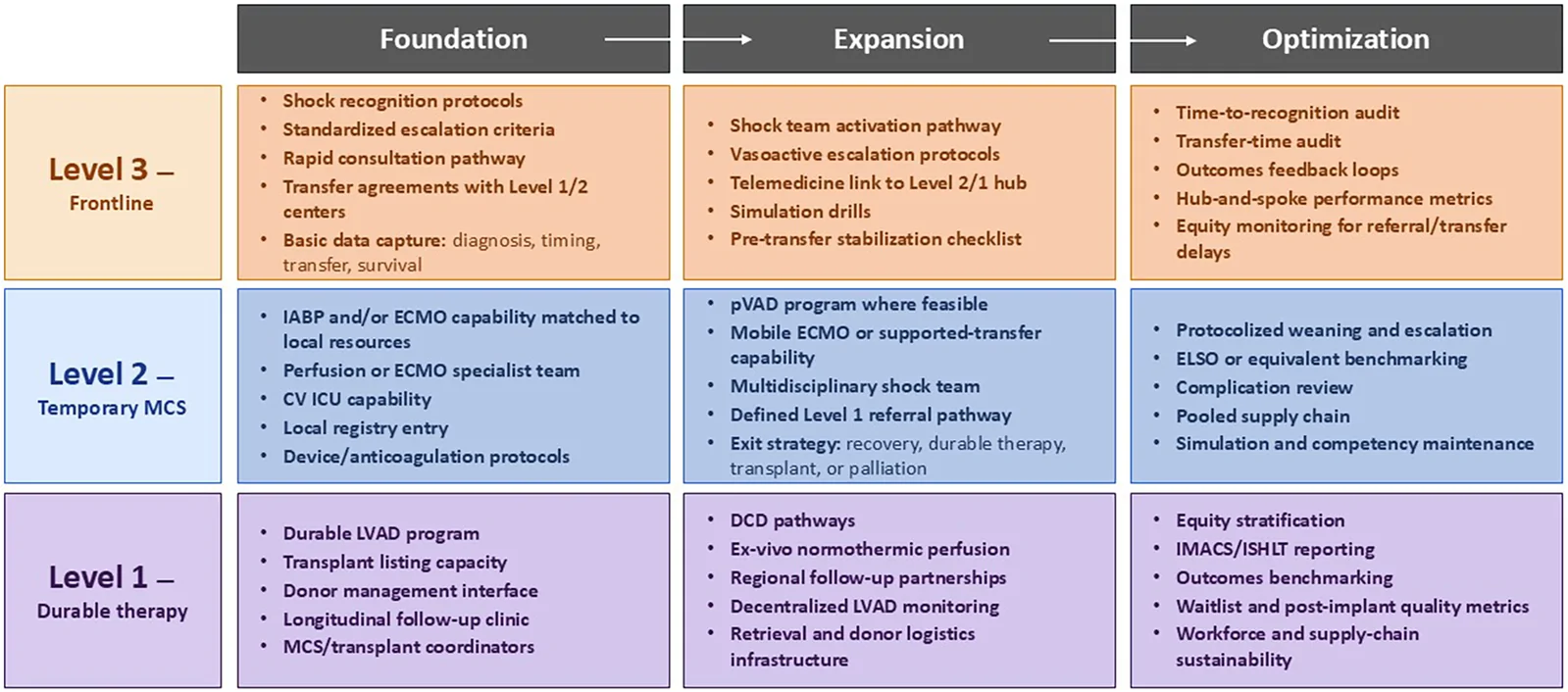
Phased implementation roadmap for expanding MCS and transplant capability. The roadmap illustrates how regional systems can mature across three center levels. Level 3 frontline centers focus on shock recognition, stabilization, consultation, transfer, and feedback loops. Level 2 centers develop temporary mechanical circulatory support capability, supported transfer pathways, and defined exit strategies. Level 1 centers provide durable mechanical circulatory support and heart transplantation, with emphasis on donor pathways, longitudinal follow-up, registry participation, benchmarking, and equity monitoring. DCD, donation after circulatory death; ECMO, extracorporeal membrane oxygenation; ELSO, Extracorporeal Life Support Organization; IABP, intra-aortic balloon pump; IMACS, International Society for Heart and Lung Transplantation Mechanically Assisted Circulatory Support Registry; ISHLT, International Society for Heart and Lung Transplantation; LVAD, left ventricular assist device; MCS, mechanical circulatory support; pVAD, percutaneous ventricular assist device.

A phased implementation roadmap can help health systems expand capability stepwise, beginning with shock recognition and transfer, progressing to temporary MCS and definitive therapy programs, and ultimately incorporating benchmarking, equity monitoring, and longitudinal quality improvement (Figure 4). Importantly, temporary MCS expansion without pathways to recovery, durable MCS, transplantation, or palliation risks reproducing the “bridge to nowhere” problem; therefore, each phase should include explicit exit strategies and referral pathways before further scale-up.

### 5.1 Technology levers

Building on the improved outcomes achieved with current-generation devices (4, 17), several emerging technologies may expand access by reducing complications, extending donor utilization, and enabling more decentralized care. Fully implantable LVAD systems using transcutaneous energy transfer remain investigational but could reduce driveline-related infection, a major source of LVAD morbidity, if long-term safety and reliability are demonstrated (88, 89). Because driveline care requires hygiene, power access, patient education, caregiver support, and reliable follow-up, reducing driveline dependence could also broaden candidacy for patients who might otherwise be considered poor candidates for durable MCS. Early first-in-human experience with wireless coplanar energy transfer has shown technical feasibility (88), although evidence remains limited and long-term durability remains uncertain (89).

Remote monitoring platforms are another technology-enabled strategy for decentralizing LVAD follow-up. These platforms may facilitate longitudinal assessment of symptoms and device-related concerns between in-person visits, supporting earlier complication recognition and extending specialist oversight to patients living far from implanting centers (90).

Ex-vivo normothermic organ perfusion represents a major technological lever for heart transplantation. By allowing functional assessment and extended preservation of donor hearts, these systems may increase use of marginal or DCD donor hearts and facilitate longer-distance organ transport (73, 91). Contemporary reviews and meta-analyses suggest that DCD heart transplantation can achieve early survival comparable to donation after brain death heart transplantation, supporting its role as an expanding donor source (73, 91).

Lower-cost and fit-for-context VAD designs may also help address access gaps in resource-limited settings, particularly if they reduce manufacturing and maintenance costs while accounting for supply-chain constraints, power reliability, maintenance capacity, and trained personnel. However, most such platforms remain early-stage or supported by limited clinical data, and access benefits will require reliable training, maintenance, and longitudinal follow-up systems (89, 92).

Temporary MCS technology is also evolving in ways that may broaden access beyond highly specialized centers. Lower-profile percutaneous devices, simplified deployment platforms, portable consoles, and modular configurations may reduce complications, shorten time to support, and expand the range of clinical scenarios in which temporary circulatory support can be deployed (23). Although temporary MCS has its place in cardiogenic shock care, many emerging lower-profile platforms remain supported primarily by early feasibility or high-risk PCI data rather than definitive cardiogenic shock outcome trials (23, 44). These technologies will improve access only if paired with training, reliable supply chains, and regional systems capable of safe patient selection, implantation, troubleshooting, and transfer.

### 5.2 System and governance levers

Regionalized systems of care are among the most important levers for expanding access to MCS and heart transplantation. Hub-and-spoke models, link frontline centers with temporary MCS-capable centers and advanced Level 1 programs through predefined consultation, transfer, and escalation pathways (44). The National Cardiogenic Shock Initiative, a prospective single-arm multicenter study, implemented a standardized AMI-cardiogenic shock protocol across 80 U.S. hospitals in 29 states, including academic and community settings, and reported 71% survival to discharge among 406 enrolled patients (93). Although the absence of a control group limits causal inference, NCSI supports the feasibility of protocolized, multidisciplinary shock care across heterogeneous practice environments.

Mobile retrieval programs represent another system lever by deploying specialized teams to stabilize patients at referring hospitals before transfer to Level 2 or Level 1 centers. Although most mature examples involve ECMO retrieval, the same principle may apply to IABP- or pVAD-supported patients when trained teams, interfacility agreements, and reliable communication infrastructure are in place (94).

For durable MCS, hub-and-spoke models may allow systems to centralize procedural expertise while decentralizing longitudinal follow-up. In Brazil, a single implanting center in São Paulo, supported by philanthropy, performed 20 LVAD implants with follow-up distributed across seven states, achieving 90% one-year and 84% two-year survival (34). Although the small sample size limits generalizability, this model illustrates how centralized implantation combined with trained regional follow-up teams may extend durable MCS access in middle-income settings.

For heart transplantation, system levers focus on improving donor identification, donor management, retrieval logistics, and organ utilization. Specialized donor care facility models, protocolized donor management goals, and broader donor acceptance practices may improve thoracic organ utilization and procurement efficiency (31, 95, 96). When legal and reimbursement structures permit, DCD pathways and ex-vivo perfusion technologies may further expand the donor pool (73, 91, 97). Differences in donor heart acceptance between Europe and the United States suggest that system-level practice patterns, not donor biology alone, influence utilization of available organs (98). Similarly, consent-policy reforms should be paired with public awareness campaigns, culturally tailored communication about brain death and donation, trained donation request processes, family engagement, transparent reporting, and investment in organ procurement infrastructure.

Across all three domains, durable access requires governance structures that align regulation, reimbursement, procurement, allocation, and referral pathways. Programs must ensure that device approval, servicing, reimbursement, inventory management, donor allocation, and retrieval logistics are coordinated before expansion (19, 34, 37, 60, 78). In resource-limited settings, pooled purchasing agreements, shared maintenance contracts, and regional referral networks may reduce costs and stockout risk, while integration of advanced cardiac surgical and heart failure services into broader health-financing frameworks may improve financial protection and long-term sustainability (19, 34, 37, 60, 78). Manufacturers, health systems, and governments may also support access through tiered pricing, service contracts, training support, and device donation or refurbishment programs where appropriate.

### 5.3 Operational sustainability and workforce models

While institutional enthusiasm and capital investment may launch MCS and transplant programs, operational sustainability determines whether they persist. These programs face recurring threats to sustainability, including 24/7 coverage requirements, maintenance of device- and procedure-related competency, and reliable logistics for devices, supplies, donor coordination, and retrieval (11, 19, 52).

Sustainable programs require robust call structures that include cannulators, perfusionists, MCS-trained intensivists, transplant cardiologists, surgeons, coordinators, and personnel capable of troubleshooting device- or donor-related emergencies. In response to workforce constraints, some centers have adopted nurse- or respiratory therapist–led ECMO specialist models. In a retrospective single-center study, implementation of a nurse-run ECMO program was associated with noninferior survival to discharge and complication rates compared with a perfusionist-run model, although generalizability may be limited by center experience, training infrastructure, and case volume (99). Staffing models should balance workforce protection with adequate procedural volume to maintain competency. Dedicated MCS and transplant coordinators should be formalized early rather than treated as informal extensions of inpatient care, because they sustain referral intake, device logistics, donor communication, protocol adherence, anticoagulation workflows, waitlist preparation, transplant coordination, discharge planning, and longitudinal follow-up (11, 49, 98).

Competency maintenance is particularly challenging because major adverse events, including pump failure, oxygenator failure, air embolism, cannulation emergencies, and donor or retrieval logistics failures, are infrequent but high-risk. Regular high-fidelity simulation can help teams rehearse cannulation, alarm troubleshooting, device failure management, crisis communication, and coordinated escalation. In a survey of ELSO centers in the United States, 46% reported having an ECMO simulation program, whereas a separate survey of ECMO credentialing practices found that simulation was included in credentialing processes at 73% of responding centers (100, 101). Simulation may be particularly important for lower-volume programs, where limited clinical exposure increases the risk of skill decay (100, 101).

Supply-chain and logistics reliability are another pillar of sustainability. Programs require preventive maintenance, replacement components, backup equipment, donor coordination, organ retrieval, and transport pathways. In lower-volume or resource-limited settings, pooled regional networks, shared backup consoles or pumps, shared perfusion capacity, pre-negotiated maintenance agreements, and coordinated retrieval or transport arrangements may reduce costs while preserving collective MCS and transplant capability (11, 34, 37, 68, 95).

### 5.4 Training and mentorship levers

Training strategies are essential for translating infrastructure into safe and sustainable clinical capacity. For temporary MCS, standardized education, simulation, and competency assessment can support the development of new shock programs while maintaining procedural quality. Training should be matched to local capability and may include IABP, pVAD, ECMO, anticoagulation, vascular access, troubleshooting, and transfer protocols. ELSO has developed structured ECMO education pathways, including modular curricula, simulation-based training, and individual practitioner certification, providing a framework that can be adapted to local resources and staffing models (100, 101).

International twinning and longitudinal mentorship partnerships with established centers offer another pathway for capacity building in resource-limited settings. Rather than relying on episodic visiting teams, successful models emphasize graduated responsibility, train-the-trainer structures, local leadership development, and progressive independence. Although direct evidence in MCS and heart transplantation remains limited, cardiac surgery capacity-building programs in settings such as Uzbekistan, Ethiopia, and Côte d'Ivoire demonstrate that sustained institutional partnerships can increase local surgical capacity over time (102–104). Remote and virtual mentorship may extend these partnerships between site visits by supporting case discussion, peri-procedural planning, post-operative troubleshooting, and longitudinal education, but should supplement rather than replace local workforce development, hands-on training, and durable institutional investment (105, 106).

### 5.5 Data, registry, and quality levers

Data infrastructure is essential for moving from isolated program development to measurable, scalable improvement. Registries allow programs to characterize case volume, patient selection, complications, survival, device utilization, and longitudinal outcomes, while also supporting benchmarking across centers and regions. For temporary MCS, the ELSO registry and Quality Reporting Platform provide mechanisms for ECMO centers to track outcomes, monitor performance, and compare results against peer benchmarks (39, 44). Other national, regional, or device-specific datasets may provide complementary information for non-ECMO temporary MCS, although coverage remains fragmented and definitions are not always harmonized. For durable MCS, the relaunch of the IMACS registry after a five-year hiatus may restore international benchmarking for durable device therapy, while EUROMACS demonstrates how registry data can be used to generate standardized outcome ratios for center-level comparison of mortality, bleeding, and stroke (87, 107).

These platforms also illustrate the need for data systems that are feasible outside high-resource settings. In many low- and middle-income countries, comprehensive registry participation may be limited by staffing, cost, data governance, and informatics infrastructure (21, 33). A pragmatic approach would prioritize a minimum dataset including indication, device type, timing of support, transfer status, complications, survival, transition to recovery or durable therapy, waitlist outcomes, and post-discharge follow-up. Such data elements would allow programs to identify gaps, support quality improvement, and advocate for resources without requiring the full data burden of mature registries. Minimum datasets should also capture equity-relevant variables, including geography, referral source, transfer status, insurance or payment mechanism where applicable, language needs, and follow-up completion, to support benchmarking across access as well as outcomes.

Data systems can also be linked to governance and accountability. Registry participation has historically been incorporated into reimbursement and coverage frameworks for LVAD programs in the United States, illustrating how payment or accreditation structures can incentivize data submission and quality. However, in resource-limited settings, mandatory reporting should be paired with technical assistance, simplified data entry, and safeguards for privacy and data ownership to avoid excluding under-resourced programs from participation. Industry partners, professional societies, and health ministries can support this process by helping standardize definitions, fund registry infrastructure, and provide technical assistance without compromising data independence, privacy or local ownership.

## 6 Limitations

This review has several limitations. First, it is a narrative, implementation-focused review rather than a systematic review, and selection bias may have influenced which studies, registries, and policy reports were emphasized. Although we prioritized contemporary peer-reviewed evidence, professional society reports, and major registry data, the available literature remains uneven across regions and therapy domains.

Second, the data sources included in this review are not fully comparable across countries or over time. Registries and annual reports differ in reporting years, case definitions, denominator selection, device classification, voluntary vs. mandatory submission, and completeness of longitudinal follow-up. As a result, international comparisons should be interpreted as indicators of broad access patterns rather than precise estimates of relative health-system performance.

Third, incomplete data from low-resource settings may underestimate unmet need and obscure non-registry-based MCS and transplant activity. Many low- and middle-income countries lack national registries for durable MCS, temporary MCS, or heart transplantation, and participation in international registries may be limited by cost, staffing, informatics infrastructure, and data-governance barriers. Finally, much of the evidence on durable LVAD therapy, temporary MCS systems, transplant outcomes, and quality improvement derives from high-income countries with established referral networks, reimbursement structures, and specialized workforces, which may limit generalizability to lower-resource settings.

## 7 Conclusion

The global landscape of MCS and heart transplantation is defined by a central paradox: device technology has advanced substantially, yet access to these therapies remains concentrated in a small number of high-resource health systems. Contemporary durable LVAD therapy has achieved meaningful long-term survival gains, and evidence for selected temporary MCS strategies continues to evolve in specific cardiogenic shock populations; however, these advances have not translated into equitable global availability. The central challenge is not innovation alone, but translation: ensuring that advances in devices, organ preservation, monitoring, and support platforms are matched by the systems needed to deliver them safely, sustainably, and equitably.

Barriers to access are interconnected across infrastructure, workforce, regulation, financing, culture, equity, and data systems. These constraints operate differently across temporary MCS, durable MCS, and heart transplantation, but they share a common root in health system capacity. Expanding access therefore requires coordinated investment across the full continuum of care, including regionalized shock systems, durable MCS referral and follow-up networks, donor management and retrieval infrastructure, sustainable workforce models, training partnerships, and registry-based quality systems.

A systems-first approach should prioritize scalable models that match local resources while preserving safety, quality, and equity. Progress will depend on pragmatic metrics that can be measured across diverse settings, including therapy use per population, transfer times, waitlist outcomes, survival, major complications, successful transition from temporary support to definitive therapy, and ongoing follow-up. Ultimately, improving global access to MCS and heart transplantation will require aligning device innovation with regional systems that can identify appropriate candidates, deliver therapy safely, measure outcomes, and sustain post-discharge care.
